# Interrogation of T Cell–enriched Tumors Reveals Prognostic and Immunotherapeutic Implications of Polyamine Metabolism

**DOI:** 10.1158/2767-9764.CRC-22-0061

**Published:** 2022-07-13

**Authors:** R. Alex Harbison, Rajeev Pandey, Michael Considine, Robert D. Leone, Tracy Murray-Stewart, Rossin Erbe, Raj Mandal, Mark Burns, Robert A. Casero, Tanguy Seiwert, Carole Fakhry, Drew Pardoll, Elana Fertig, Jonathan D. Powell

**Affiliations:** 1 Department of Otolaryngology, Johns Hopkins University School of Medicine, Baltimore, Maryland.; 2 Department of Otolaryngology Oncology, Johns Hopkins University School of Medicine, Baltimore, Maryland.; 3 Department of Otolaryngology Human Genetics, Johns Hopkins University School of Medicine, Baltimore, Maryland.; 4 Department of Biomedical Engineering, Sidney Kimmel Comprehensive Cancer Center, Johns Hopkins University School of Medicine, Baltimore, Maryland.; 5 Aminex Therapeutics, Kirkland, Washington.; 6 The Bloomberg-Kimmel Institute for Cancer Immunotherapy, Johns Hopkins University School of Medicine, Baltimore, Maryland.

## Abstract

Metabolic features of the tumor microenvironment (TME) antagonize antitumor immunity. We hypothesized that T cell–infiltrated (Thi) tumors with a known antigen should exhibit superior clinical outcomes, though some fare worse given unfavorable metabolic features leveraging T cell–infiltrated (Thi), human papillomavirus–related (HPV^+^) head and neck squamous cell carcinomas (HNSC) to test this hypothesis. Expression of 2,520 metabolic genes was analyzed among Thi HPV^+^ HNSCs stratified by high-risk molecular subtype. RNA sequencing (RNA-seq) data from The Cancer Genome Atlas (TCGA; 10 cancer types), single-cell RNA-seq data, and an immunotherapy-treated melanoma cohort were used to test the association between metabolic gene expression and clinical outcomes and contribution of tumor versus stromal cells to metabolic gene expression. Polyamine (PA) metabolism genes were overexpressed in high-risk, Thi HPV^+^ HNSCs. Genes involved in PA biosynthesis and transport were associated with T-cell infiltration, recurrent or persistent cancer, overall survival status, primary site, molecular subtype, and *MYC* genomic alterations. PA biogenesis gene sets were associated with tumor-intrinsic features while myeloid cells in HPV^+^ HNSCs were enriched in PA catabolism, regulatory, transport, putrescine, and spermidine gene set expression. PA gene set expression also correlated with IFNγ or cytotoxic T-cell single-sample gene set enrichment analysis (ssGSEA) scores across TCGA tumor types. PA transport ssGSEA scores were associated with poor survival whereas putrescine ssGSEA scores portended better survival for several tumor types. Thi melanomas enriched in PA synthesis or combined gene set expression exhibited worse anti-PD-1 responses. These data address hurdles to antitumor immunity warranting further investigation of divergent PA metabolism in the TME.

**Significance:**

Despite the presence of tumor-infiltrating lymphocytes and antigen, antitumor immunity is often insufficient in tumor control. We leverage HPV-related head and neck cancers to identify metabolic challenges to antitumor immune responses. PA metabolism is associated with tumor-intrinsic features while the myeloid compartment exhibits enriched PA regulatory gene expression.

## Introduction

Features promoting antitumor immunity include the presence of cytotoxic tumor-infiltrating lymphocytes (TIL) and antigen (e.g., viral, tumor neoantigens; refs. 1–4). However, not all immune cell–infiltrated, immunogenic tumors exhibit favorable antitumor immunity. The tumor microenvironment (TME) diminishes antitumor T-cell function through recruitment of tolerogenic cell types, nutrient depletion, and the creation of an acidic, hypoxic microenvironment (5). Metabolism affects immune checkpoint inhibition (ICI) response. ICI may be most effective in highly glycolytic tumors (6). Therefore, we hypothesized that metabolic adaptations diminish antitumor immunity in an otherwise favorable TME.

To test this hypothesis, we selected a cohort of human papillomavirus (HPV)-associated head and neck squamous cell carcinomas (HNSC) characterized by CD8^+^ T-cell infiltration and virus-derived tumor-associated antigens (4). By leveraging the immune characteristics of these tumors, we surmised that tumor-intrinsic or -extrinsic features of the TME diminish the antitumor immune response in some, but not all tumors, focusing on metabolic features of the TME. We leveraged genomic atlases to test our hypothesis. We validated our findings across 10 different cancers from The Cancer Genome Atlas (TCGA) and immunotherapy-treated melanomas.

## Materials and Methods

### Clinical Data Collection

TCGA level 1 clinical data were abstracted from FireBrowse (http://firebrowse.org/). Data for the HNSC samples were derived from: gdac.broadinstitute.org_HNSCC.Merge_Clinical.Level_1.2016012800.0.0/HNSCC.clin.merged.txt. The HPV status was identified using the variable: patient.hpv_test_results.hpv_test_result.hpv_status (levels: positive, negative, indeterminate).

### RNA Sequencing and Alignment

Sequencing and alignment of TCGA data have been described previously (https://docs.gdc.cancer.gov/Data/Bioinformatics_Pipelines/Expression_mRNA_Pipeline/). TCGA RSEM expression data were obtained through FireBrowse. File names from each cancer site are documented in Supplementary Table S17.

### RNA Expression Data Preparation and Analysis

RSEM expression data were extracted and preprocessed by excluding genes with zero reads across tumors. Genes with detectable reads in at least 50% of samples were included. We used the variance stabilizing transformation and normalization function in DESeq2 (RRID: SCR_015687) to normalize data for use in downstream analyses (7). Differential expression (DE) analysis was performed using DESeq2 on RSEM data rounded to the nearest integer. Network analysis was performed using Shiny GAM: integrated analysis of genes and metabolites (https://artyomovlab.wustl.edu/shiny/gam/; ref. 8). Hierarchical clustering was performed with the ComplexHeatmap R package (RRID: SCR_017270) using the “ward.D” clustering method and “Pearson” distance on the rows and columns (9).

### T-Cell Receptor Diversity Analysis

T-cell receptor (TCR) diversity was assayed and reported in the Supplementary Data of Thorsson and colleagues (10) We used the Shannon entropy and Richness variables from TCGA HNSC data to test the relationship between polyamine (PA) pathway gene set expression and TCR diversity.

### Single-Sample Gene Set Enrichment Analysis

Single-sample gene set enrichment analysis (ssGSEA; v10.0.3) was implemented in GenePattern (RRID:SCR_003201) to estimate PA pathway and immune gene set scores (Supplementary Table S1; ref. 11). Default parameters were used with rank normalization for all ssGSEAs.

### Cellular Abundance Estimates from Bulk RNA-sequencing Data

We utilized CIBERSORT in “Impute Cell Expression” mode on TCGA transcripts per million (TPM) RNA sequencing (RNA-seq) data from patients with HNSC to infer relative cellular proportions. For the CIBERSORT analysis, we used a HNSC reference single-cell (sc) RNA-seq dataset (12) in TPM normalization space to define proportions of tumor cells, macrophages, fibroblasts, CD8^+^, and CD4^+^ T cells in bulk RNA-seq data. TCGA HNSC TPM RNA-seq data were used for this analysis to keep the bulk RNA-seq and reference matrix in the same normalization space per CIBERSORT recommendations.

### T-Cell Infiltration Stratification

T-cell infiltration scores from ssGSEA were generated using CD8^+^ T-cell and cytotoxic T cell (CTL) gene sets described by Bindea and colleagues (ref. 13; Supplementary Table S1). We scaled ssGSEA scores from each gene set. Samples were dichotomized [T cell–enriched (Thi) vs. T cell–depleted (Tlo)] using an upper quartile cutoff of ssGSEA scores for both CTL and CD8^+^ T-cell gene sets (Supplementary Table S2). Tumors in the highest ssGSEA score quartile for either the CD8^+^ T cell or CTL signature were categorized as Thi and the remainder Tlo (Supplementary Table S2). ssGSEA stratification of T-cell infiltration status was consistent with inferred CD8^+^ T-cell abundance from computational microdissection with CIBERSORT (Wilcoxon rank-sum of CD8^+^ T-cell abundance between Thi and Tlo, *P* < 0.001; Supplementary Fig. S1; ref. 14). ssGSEA stratification was used for all downstream analyses for (i) comparability with other studies using the Bindea immune cell gene sets to infer immune cell responses; and (ii) to use a consistent, gene set–driven approach, agnostic to the tissue of origin.

### Metabolic Gene Curation

We defined a set of 2,520 genes implicated in metabolism using gene sets from Broad Institute's Molecular Signature Database (MSigDB; ref. 15) and Shaul and colleagues (16).

### Survival Analysis

We utilized univariate Cox regression analysis to test associations between PA pathway scores and risk of mortality.

### Molecular Subtype Classification

To define molecular subtypes using TCGA HNSC expression data, we utilized an R script kindly provided by the Fertig and Seiwert labs based on prior work which implements a correlation-based nearest centroid technique (17). Subtypes were assigned for the entire TCGA HPV^+^ and HPV^−^ HNSC dataset including basal, classical, or mesenchymal subtypes.

### HPV Integration

Using data from Parfenov and colleagues, we assigned TCGA HPV^+^ HNSC viral integration status (18).

### Tumor Mutation Burden

Tumor mutation burden (TMB) data from TCGA MC3 (19) were extracted using maftools (20). We defined high TMB as ≥10 mutations/megabase pair (Mbp) and low TMB as <10 mutations/Mbp.

### scRNA-Seq Analysis

HPV^+^ HNSC TIL data from Cillo and colleagues (21) were downloaded, preprocessed, normalized, and scaled using Seurat (v4.0.1; refs. 22, 23). Data were mapped onto a single-cell reference dataset to identify immune cell subsets (22) which were used to evaluate expression of PA pathway enzymes across cell types. The “AddModuleScore” function was used to determine PA gene set (i.e., “module”) scores using the gene sets defined below. A similar process was used for HPV^−^ HNSC scRNA-seq data which were TPM normalized and were preprocessed, log_2_ transformed, and scaled (12).

### Statistical Analysis

R programming software (version 3.6.1) was used for statistical analyses (24). Kruskal–Wallis or Wilcoxon tests were used to compare data distributions between more than two groups or two groups, respectively, for non-normally distributed data. *χ*^2^ tests were used to evaluate independence between groups with expected cell counts ≥5. Fisher exact tests were used to test for independence with any expected cell count <5. To account for multiple hypothesis testing, the FDR was controlled using the method of Benjamini and Hochberg. Pearson correlation analyses were performed in R using the WGCNA software package (RRID:SCR_003302; ref. 25). An alpha of 0.05 was used as a threshold for statistical significance, except in the case of multiple hypothesis testing where a *q* value of 0.25 was used. *, *P* ≤ 0.05; **, *P* ≤ 0.01; ***, *P* ≤ 0.001; ****, *P* ≤ 0.0001; ns, *P* > 0.05.

### Data Availability Statement

TCGA expression data are available from FireBrowse (http://firebrowse.org/) with the filenames defined in Supplementary Table S17. Accession numbers for data accessed through the NCBI Gene Expression Omnibus include GSE91061 (26), GSE10322 (12), and GSE139324 (21). Source code is available online through the GitHub repository: https://github.com/alexharbison/polyamines_immunometabolism_cancer.git (DOI: 10.5281/zenodo.4959622).

### Ethics Approval and Consent to Participate

Data were obtained from publicly available databases.

## Results

### Survival Among T Cell–enriched, Antigen-driven HPV^+^ HNSC

**FIGURE 1:**
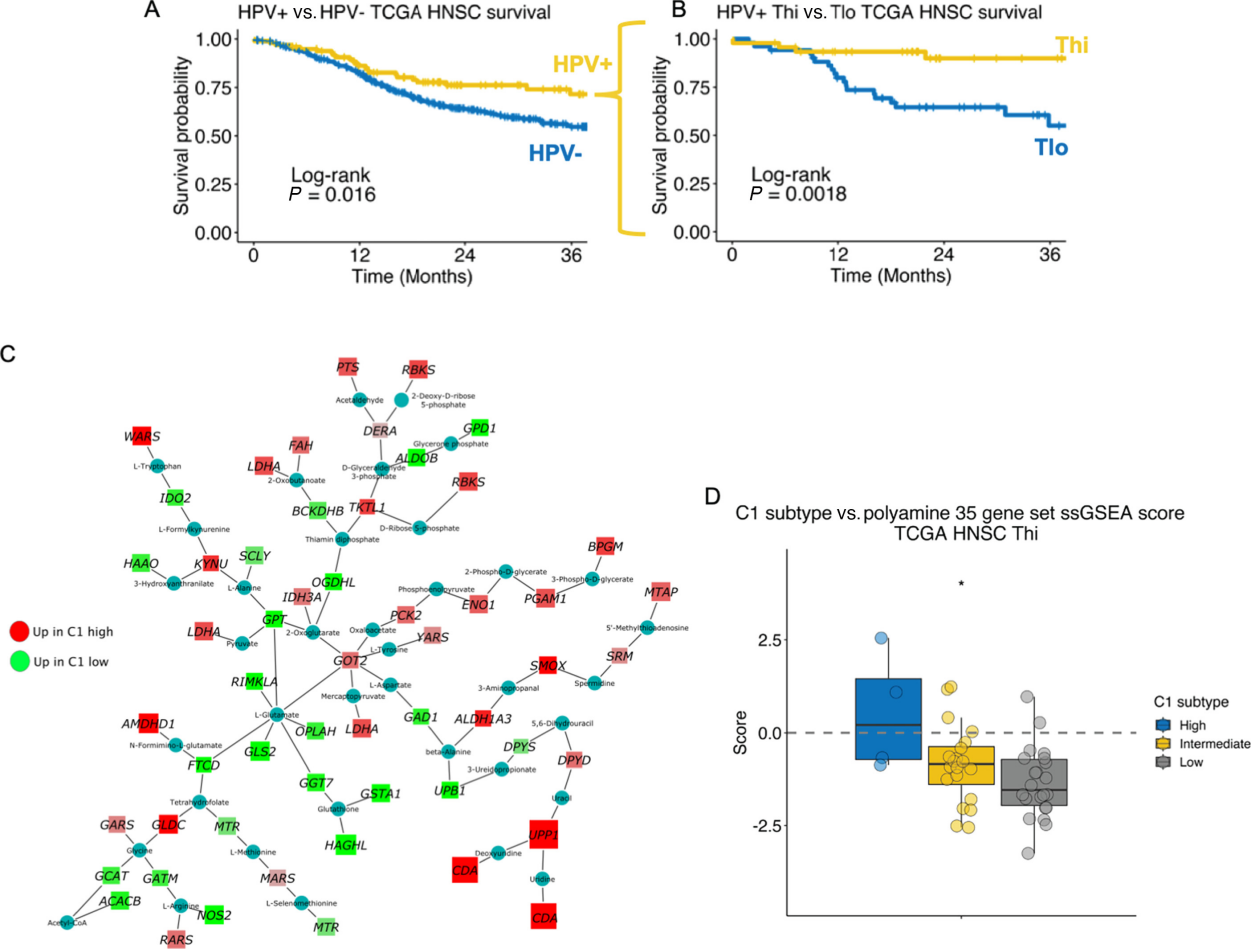
PA metabolism genes are differentially expressed between T cell–infiltrated HPV-related (HPV^+^) HNSCs stratified by a prognostic molecular gene signature. **A,** Survival among TCGA HNSC stratified by HPV status [HPV^+^, *n* = 97; HPV^−^ (carcinogen-driven), *n* = 421]. **B,** HPV^+^ HNSC tumors were stratified by T-cell infiltration as inferred by ssGSEA CD8^+^ T-cell and cytotoxic lymphocyte gene set scores. Survival based on T-cell infiltration status is shown. **C,** Metabolic gene transcriptional network analysis comparing TCGA HPV^+^ Thi HNSCs based on the high-risk HPV^+^ HNSC C1 gene set DE results (C1 high vs. C1 low). Red nodes, gene expression enriched in C1 high tumors. Green nodes, gene expression enriched in C1 low HPV^+^ HNSCs. **D,** ssGSEA scores from a curated set of 35 genes involved in PA metabolism among TCGA HPV^+^ Thi HNSCs stratified by C1 gene set expression strata divided into high, intermediate, and low. *, *P* ≤ 0.05.

We stratified TCGA HPV^+^ HNSCs into high (Thi) and low (Tlo) T-cell infiltration. Of the HPV^+^ HNSCs, 47% (46/97) were Thi whereas only 31% (130/420) HPV^−^ HNSCs were Thi (*χ*^2^ test, *P* = 0.002). Patients with HPV^+^ HNSC had better survival than carcinogen-driven (HPV^−^) HNSCs (Fig. 1A). Among the HPV^+^ HNSCs, 3-year survival probability was greater for Thi [0.90, 95% confidence intrerval (CI): 0.81–1.0] than Tlo tumors (0.55, 95% CI: 0.40–0.75; log-rank test, *P* = 0.0018; Fig. 1B). In contrast, HPV^−^ HNSCs did not have better 3-year survival when stratified by T-cell status (Tlo: 0.53, 95% CI: 0.46–0.60; Thi: 0.59, 95% CI: 0.51–0.70; log-rank test, *P* = 0.42).

### Metabolic Genes Enriched Among High-risk, HPV^+^ T Cell–infiltrated HNSCs

To identify metabolic features impairing the antitumor immune response in an otherwise favorable viral antigen positive, T cell–enriched TME, we performed a metabolism focused transcriptional network analysis (8) comparing TCGA HPV^+^ Thi HNSCs stratified by a high-risk HPV^+^ HNSC gene signature (i.e., C1; ref. 27). We hypothesized that T cell–infiltrated HPV^+^ C1 HNSCs exhibit a poor prognosis secondary to aberrant metabolic features. The C1 signature is driven primarily by tumor cell–intrinsic expression. Notably, immune-related signatures are not differentially expressed between the C1 (worse prognosis) and C2 (better prognosis) HPV^+^ HNSC subsets. The HPV^+^ C1 signature demonstrates an intermediate gene expression profile between HPV^−^ and HPV^+^ tumors with better survival (i.e., HPV^+^ C2) and has lower expression of the HPV E1^E4 splicing isoform, a feature associated with resistance to radiation.

To identify metabolic gene network differences between TCGA HPV^+^ Thi HNSCs stratified by the C1 signature, we generated a score across tumors taking the cumulative expression across C1 genes (*CDA*, *DFNA5*, *KRT14*, *MDFI*, *UPP1*, *DPF1*, *RGS20*, *TUBB3*, *PPP1R14B*, *RHOD*, *CPA4*, *PNLIPRP3*) for each sample and stratifying them into high, intermediate, and low strata. The distribution of C1 strata by T-cell infiltration status is shown in Table 1. Nine percent of Thi tumors and 55% of Tlo tumors were C1 high (*χ*^2^ test, *P* < 0.001).

**TABLE 1:** Distribution of TCGA HPV^+^ Thi and Tlo HNSCs by clinical and genomic features

T-cell status			
Variable	Thi, *N* = 46^a^	Tlo, *N* = 51^a^	*P*
C1 score			<0.001^b^
High	4/46 (8.7%)	28/51 (55%)	
Intermediate	20/46 (43%)	12/51 (24%)	
Low	22/46 (48%)	11/51 (22%)	
Group			<0.001^b^
Basal	0/46 (0%)	15/51 (29%)	
Classical	8/46 (17%)	30/51 (59%)	
Mesenchymal	38/46 (83%)	6/51 (12%)	
Site			<0.001^c^
Hypopharynx	1/46 (2.2%)	4/51 (7.8%)	
Larynx	2/46 (4.3%)	4/51 (7.8%)	
Oral cavity	7/46 (15%)	25/51 (49%)	
Oropharynx	36/46 (78%)	18/51 (35%)	
*PIK3CA*			0.44^b^
Alteration^d^	36/46 (78%)	44/51 (86%)	
No alteration	10/46 (22%)	7/51 (14%)	
*AKT1*			0.44^b^
Alteration^d^	7/46 (15%)	12/51 (24%)	
No alteration	39/46 (85%)	39/51 (76%)	
*MTOR*			0.16^b^
Alteration^d^	9/46 (20%)	4/51 (7.8%)	
No alteration	37/46 (80%)	47/51 (92%)	
*MYC*			0.035^b^
Alteration^d^	19/46 (41%)	33/51 (65%)	
No alteration	27/46 (59%)	18/51 (35%)	

^a^Statistics presented: *n*/*N* (%).

^b^ *χ* ^2^ test of independence.

^c^Fisher exact test.

^d^Alteration defined a presence of mutation, copy-number gain or amplification, or homozygous deletion.

Focusing our analysis on 2,520 metabolism-related genes derived from MSigDB (1,617 genes total; refs. 15, 28) and Shaul and colleagues (ref. 16; 903 additional genes; Supplementary Table S3), DE analysis comparing HPV^+^ Thi C1 high (C1hi; *N* = 4) versus C1 low (C1lo; *N* = 22) tumors revealed 935 metabolic genes upregulated in the HPV^+^ Thi C1hi HNSCs (Supplementary Table S4). Database for Annotation, Visualization and Integrated Discovery (DAVID) analysis demonstrated enrichment of genes involved in mitogenic signaling (*TSC2*, *MAPK1*, *INSR*, *PIK3CG*) plus lipid (*INPP5A*, *LPL*), central carbon (*IDH2*, *SDHB-D, HK1*, *HK2*, *LDHA*, *LDHC*), arginine and proline metabolism (*SMOX*, *ARG2*, *NOS2*, *AMD1*, *SRM*, *AGMAT*, *PRODH*; Supplementary Table S5) among the HPV^+^ Thi C1 high tumors. DE results were used as input for network analysis focused on identification of the most differing metabolic subnetworks between C1hi versus C1lo tumors (Fig. 1C). Consistent with the DAVID pathway-level analysis, we observed a subnetwork of PA metabolism gene enrichment among the C1hi tumors including *SMOX*, *SRM*, and *MTAP*. Notably, this PA subnetwork illustrated a close association with lactate (*LDHA*), kynurenine (*KYNU*, *IDO2*), tryptophan (*WARS*), beta-alanine (*UPB1*), and ⍺-ketoglutarate (*IDH3*) metabolism (Fig. 1C; Supplementary Fig. S2). Quantification of PA metabolism gene expression was performed using ssGSEA as a function of C1 expression strata. A set of 35 curated genes involved in PA metabolism and transport were quantified across TCGA HPV^+^ Thi HNSCs using ssGSEA demonstrating lower PA ssGSEA scores among the C1lo tumors relative to the C1 intermediate or C1hi tumors (Fig. 1D).

We also compared DE among HPV^+^ Thi tumors based on their molecular subtype (classical vs. immune/mesenchymal; Supplementary Table S6). Among HPV^+^ HNSCs, the immune/mesenchymal subtype has a better prognosis than classical subtype tumors (17). The immune/mesenchymal subtype is characterized by immune markers (e.g., *CD8A*, *ICOS*) and mesenchymal markers (e.g., *VIM*, *S100A4*). The most distinctive transcriptional feature of the classical subtype in both HPV^+^ and HPV^−^ HNSCs is enrichment for PA metabolism gene expression (17). Molecular subtype across TCGA HNSCs was defined using a correlation-based, nearest centroid classification approach (17). Table 1 shows the distribution of molecular subtypes given T-cell status across HPV^+^ HNSCs. Eighty-three percent of Thi tumors and 12% of Tlo tumors were classified as mesenchymal (*χ*^2^ test, *P* < 0.001; Table 1). No HPV^+^ Thi tumors were classified as basal. The basal subtype is characterized by a predominance of HPV^−^ HNSCs, hypoxia, and EGFR/HER signaling (17). The remainder of Thi tumors were classified as classical (*N* = 8). DE analysis demonstrated 438 metabolic genes upregulated in the classical tumors. DAVID pathway analysis revealed upregulation of genes involved in mitogenic signaling (*GSK3B*, *TSC2*, *BRAF*, *PIK3CD*) in addition to lipid (*INPP4A*, *PLA2G2A*, *LPIN2*), purine (*NT5E*, *ENTPD1*, *NME3*), central carbon (*IDH1*, *HK2*, *ACAT1*), arginine, and proline metabolism (*ARG2*, *NOS1*, *PRODH*; Supplementary Table S7). Given these findings and prior metabolomic data demonstrating enrichment of PAs in HNSC tissues (29, 30), we next sought to test the relationship between PA expression and clinical and tumor genomic features.

### PA Synthesis and Transport Genes are Associated with Worse Clinical and Molecular Features Among Patients with HPV^+^ HNSC

**FIGURE 2:**
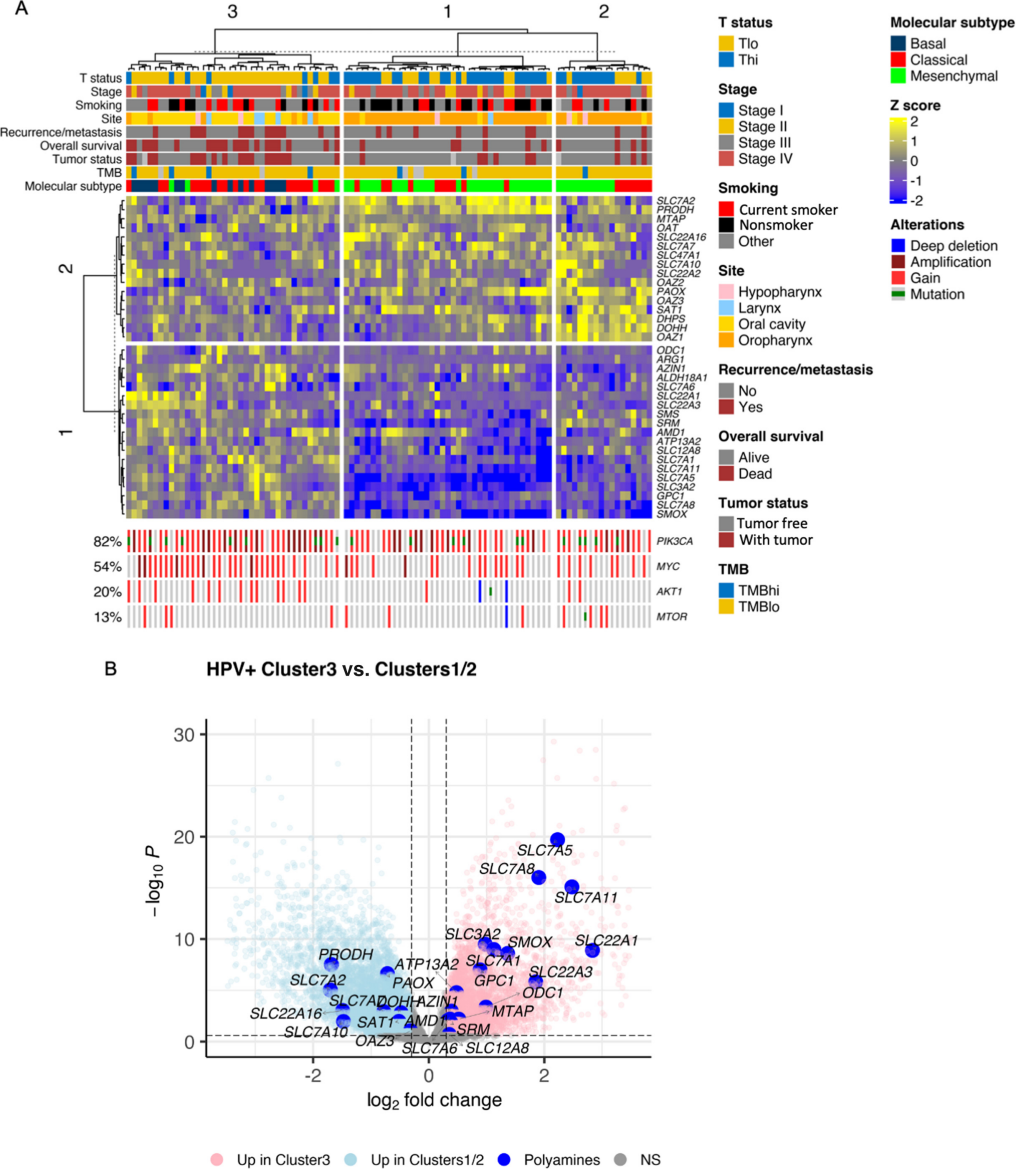
PA synthesis and transport genes are associated with worse clinical and molecular features among patients with HPV^+^ HNSC. **A,** Heatmap illustrates hierarchical clustering of scaled variance transformed RSEM counts for PA synthesis, catabolism, hypusination, and transport genes among TCGA HPV^+^ HNSCs (*n* = 97). Clinical and molecular features are annotated in the bars above the heatmap. Genomic annotations are plotted at the bottom of the heatmap for the genes shown. Rows represent unique genes. Columns represent unique patients. Hierarchical clustering was performed across columns and rows. *Stage*, AJCC 7th edition clinical stage. *TMB (tumor mutation burden)*: *TMBhi*, ≥10 mutations/Mbp; *TMBlo*, <10 mutations/Mbp. **B,** Volcano plot demonstrating DE analysis results comparing cluster 3 with clusters 1 and 2 based on the hierarchical clustering results in **A**. *Large blue dots*, PA metabolism genes. NS, nonsignificant.

To characterize the extent to which PA metabolism genes are related to clinical and molecular features among HPV^+^ HNSCs, we performed hierarchical clustering of gene expression using the 35 PA metabolism-related genes introduced above. Hierarchical clustering revealed three sample clusters (Fig. 2A, *column clusters*). Oropharyngeal tumors made up most of the cohort (54/97) followed by oral cavity cancers (32/97; Table 2). Clusters 1 and 2 consisted of 87% and 78% oropharyngeal tumors versus cluster 3 which was enriched in oral cavity tumors (68%; Fisher exact test, *P* < 0.001). Clusters 1 and 2 were predominantly Thi (69% (27/39) and 61% (11/18), respectively; Fig. 2A; Table 2). Cluster 3 tumors were mainly Tlo (80% (32/40)). The distribution of Thi and Tlo tumors by primary site is shown in Table 1. Oropharyngeal tumors comprised 78% of Thi tumors and 35% of Tlo tumors. Tumor stage did not differ between clusters nor did smoking status (Fig. 2A; Table 2).

**TABLE 2:** Clinical and molecular characteristics of TCGA HPV^+^ HNSC clusters based on polyamine metabolism gene expression

Cluster				
Variable	Cluster 1, *N* = 39^a^	Cluster 2, *N* = 18^a^	Cluster 3, *N* = 40^a^	*P* ^b^
Tstatus				<0.001
Thi	27/39 (69%)	11/18 (61%)	8/40 (20%)	
Tlo	12/39 (31%)	7/18 (39%)	32/40 (80%)	
Stage				0.67
Stage I	0/39 (0%)	1/18 (5.6%)	3/40 (7.5%)	
Stage II	6/39 (15%)	1/18 (5.6%)	5/40 (12%)	
Stage III	6/39 (15%)	3/18 (17%)	7/40 (18%)	
Stage IV	27/39 (69%)	13/18 (72%)	25/40 (62%)	
Recurrence/metastasis	7/39 (18%)	2/18 (11%)	13/40 (32%)	0.18
Tumor status				0.007
Tumor free	32/38 (84%)	14/17 (82%)	21/39 (54%)	
With tumor	6/38 (16%)	3/17 (18%)	18/39 (46%)	
(Missing)	1	1	1	
Overall survival				0.003
Alive	33/39 (85%)	14/18 (78%)	20/40 (50%)	
Dead	6/39 (15%)	4/18 (22%)	20/40 (50%)	
Smoking				0.14
Current smoker	8/39 (21%)	4/17 (24%)	13/40 (32%)	
Nonsmoker	17/39 (44%)	3/17 (18%)	8/40 (20%)	
Other	14/39 (36%)	10/17 (59%)	19/40 (48%)	
(Missing)	0	1	0	
Site				<0.001
Hypopharynx	1/39 (2.6%)	2/18 (11%)	2/40 (5.0%)	
Larynx	1/39 (2.6%)	0/18 (0%)	5/40 (12%)	
Oral cavity	3/39 (7.7%)	2/18 (11%)	27/40 (68%)	
Oropharynx	34/39 (87%)	14/18 (78%)	6/40 (15%)	
TMB category				0.88
TMBhi	2/35 (5.7%)	1/18 (5.6%)	4/38 (11%)	
TMBlo	33/35 (94%)	17/18 (94%)	34/38 (89%)	
(Missing)	4	0	2	
Group				<0.001
Basal	0/39 (0%)	0/18 (0%)	15/40 (38%)	
Classical	10/39 (26%)	7/18 (39%)	21/40 (52%)	
Mesenchymal	29/39 (74%)	11/18 (61%)	4/40 (10%)	
*PIK3CA*				0.056
Alteration	28/39 (72%)	15/18 (83%)	37/40 (92%)	
No alteration	11/39 (28%)	3/18 (17%)	3/40 (7.5%)	
*AKT1*				0.039
Alteration	4/39 (10%)	2/18 (11%)	13/40 (32%)	
No alteration	35/39 (90%)	16/18 (89%)	27/40 (68%)	
*MTOR*				0.20
Alteration	4/39 (10%)	5/18 (28%)	4/40 (10%)	
No alteration	35/39 (90%)	13/18 (72%)	36/40 (90%)	
*MYC*				0.007
Alteration	15/39 (38%)	8/18 (44%)	29/40 (72%)	
No alteration	24/39 (62%)	10/18 (56%)	11/40 (28%)	

^a^Statistics presented: *n*/*N* (%).

^b^Statistical tests performed: *χ*^2^ test of independence; Fisher exact test.

Recurrent/Metastatic (R/M), tumor status, and overall survival status were worse in cluster 3. Thirty-two percent of cluster 3 subjects experienced R/M compared with 18% and 11% of clusters 1 and 2, respectively (Fisher exact test, *P* = 0.18; Fig. 2A; Table 2). Cluster 3 had a higher proportion of patients with recurrent or persistent tumors [“with tumor”; 46% (18/39)] than clusters 1/2 subjects [16% (6/38) and 18% (3/17); Fisher exact test, *P* = 0.007; Table 2]. Clusters 1/2 subjects experienced a lower frequency of deaths over the 3-year follow-up interval [15% (6/39) and 22% (4/18), respectively] than cluster 3 subjects [50% (20/40); *χ*^2^ test, *P* = 0.003; Table 2]. R/M was not different between clusters among Thi (Fisher exact test, *P* = 0.4) or Tlo subsets (Fisher exact test, *P* = 0.46; Supplementary Table S8). Thi tumors had a higher percentage of recurrent or persistent tumors in cluster 1 and 3 (22% and 25%, respectively) compared with cluster 2 (0%; Fisher exact test, *P* = 0.23; Supplementary Table S8). Clusters 1 and 2 exhibited upregulation of PA regulatory genes (i.e., *SAT1*, *OAZ1–3*; Fig. 2B). These results suggest that PA metabolism gene expression varies by CTL infiltration and is associated with clinical features and primary site.

Next, we evaluated the relationship between TMB and molecular subtype (i.e., immune/mesenchymal vs. classical; ref. 17) with T-cell infiltration among sample clusters. High TMB was present in 11% (4/38), 5.6% (1/18), and 5.7% (2/35) of tumors in clusters 3, 2, and 1, respectively (Fisher exact test, *P* = 0.88; Table 2). Molecular subtype analysis demonstrated a high rate of mesenchymal tumors in clusters 1 and 2 [74% (29/39) and 61% (11/18), respectively] compared with cluster 3 which was enriched in classical subtype tumors [52% (21/40); Fisher exact test, *P* < 0.001; Fig. 2A; Table 2]. While the mesenchymal subtype may be associated with the degree of cancer-associated fibroblasts (12), there was a strong association between mesenchymal subtype and Thi status (Table 1).

Genomic alterations including copy-number gain, amplification, homozygous deletion, and mutations of *PIK3CA, AKT1*, *MTOR*, and *MYC* were analyzed given their role in regulating PA metabolism. *AKT1* and *MYC* were altered more frequently in cluster 3 (32% and 72%, respectively) compared with clusters 1 and 2 (10% and 38% vs. 11% and 44%, respectively; *AKT1*: Fisher exact test, *P* = 0.039; *MYC*: *χ*^2^ test, *P* = 0.007; Table 2). *PIK3CA* was altered in 72%, 83%, and 92% of cases in clusters 1, 2, and 3, respectively (Fisher exact test, *P* = 0.056; Table 2). There was no difference in *MYC* alterations between clusters in the Thi tumors (Fisher exact test, *P* = 0.84; Supplementary Table S8). *MTOR* alterations were more frequent among Thi tumors in clusters 2 (45%) and 3 (25%) compared with cluster 1 (7%; Fisher exact test, *P* = 0.016; Supplementary Table S8). *PIK3CA* and *AKT1* did not vary between clusters. Genomic alterations did not vary by T-cell status, except for *MYC* which was enriched in Tlo tumors (65% vs. 41% of Thi tumors; *χ*^2^ test, *P* = 0.035; Table 1).

Genewise clustering analysis was performed. PA genes clustered into two groups. Group 1 included genes involved in synthesis (*ODC1*, *ARG1*, *SMS*, *SRM*, *AMD1*) and transport, and Group 2 included genes involved in the regulation of PA transport and ODC activity (*OAZ1*, *OAZ2*, *OAZ3*), hypusination (*DOHH*, *DHPS*), and PA transport (Fig. 2A). Group 1 PA genes were differentially enriched in cluster 3 tumors (except *ARG1*, *ALDH18A1*, and *SMS*) and downregulated in clusters 1 and 2 (Fig. 2B; Supplementary Table S9). In comparison, PA synthesis genes (*SRM*, *AMD1*, *ARG1*), transporters (e.g., *SLC3A2* and *GPC1*), and *SMOX* were enriched in HPV^+^ Tlo relative to HPV^+^ Thi HNSCs (Supplementary Fig. S3A; Supplementary Table S10). HPV^−^ HNSCs were also differentially enriched in PA synthesis genes (*SRM*, *ODC1*), transporters (*SLC3A2*, *GPC1*, *SLC7A1*), and *SMOX* expression compared with HPV^+^ HNSC (Supplementary Fig. S3B; Supplementary Table S10). Taken together, these analyses reveal a propensity for T-cell infiltration among HPV^+^ oropharyngeal squamous cell carcinomas whereas HPV^+^ oral cavity squamous cell carcinomas appear to be largely T-cell deficient and enriched in the more aggressive basal and classical molecular phenotypes. Strikingly, HPV^+^ HNSCs clustered into T cell–enriched and T cell–deficient clusters based on expression of a set of 35 curated PA metabolism genes. Whether this reflects the role of PAs in T-cell metabolic function or tumor cell–intrinsic gene expression features which influence the extent of T-cell infiltration remains unknown. Therefore, our next objective was to evaluate the extent to which tumor cells and stromal cells contribute to PA metabolism gene expression.

### PA Gene Expression and Tumor-intrinsic Features

First, we tested whether PA gene expression is a function of tumor-intrinsic features (i.e., molecular subtype, HPV status, HPV integration, or TMB). We defined PA biogenesis–specific gene sets based on prior knowledge (Table 3; ref. 31). We included broader PA biogenesis–defined gene sets (i.e., synthesis, catabolism, regulatory, and transport) as well as metabolite specific gene sets (i.e., putrescine, spermidine, and hypusine) given the unique cellular functions of these respective metabolites. *PAOX* was included in both the putrescine and spermidine synthesis gene sets as it oxidizes N-acetylated PAs to generate putrescine and spermidine. The *SAT1* gene product catalyzes PA acetylation. *AMD1* is included in the spermidine synthesis gene set as it encodes S-adenosylmethionine decarboxylase which produces the aminopropyl donor, S-adenosylmethionine, necessary for spermidine synthesis. Hypothesizing that PA metabolites can be immunosuppressive, the “combined” gene set was informatically and empirically defined using PA genes that were negatively correlated with both Bindea cytotoxic lymphocyte gene set (CTL) and REACTOME IFNγ gene set ssGSEA scores across multiple TCGA T cell–infiltrated tumor types (Supplementary Figs. S4A and S4B; ref. 32). The transporters included have been previously reported as putative PA transporters (31, 33–44).

**TABLE 3:** PA metabolism gene sets

Pathway	Description	Genes
*Synthesis*	Polyamine biosynthesis.	*ODC1, SRM, SMS, AMD1*
*Catabolism*	Polyamine catabolism.	*SAT1, PAOX, SMOX*
*Regulatory*	Regulation of biosynthesis and transport.	*OAZ1, OAZ2, OAZ3*
*Transport*	Putative polyamine transporters.	*ATP13A2, GPC1, SLC3A2, SLC7A2, SLC7A5, SLC7A6, SLC7A7, SLC7A8, SLC7A10, SLC7A11, SLC12A8, SLC18B1, SLC22A1, SLC22A2, SLC22A3, SLC22A16, SLC47A1*
*Putrescine*	Putrescine biogenesis.	*PAOX, SAT1, ODC1*
*Spermidine*	Spermidine biogenesis.	*PAOX, SAT1, SRM, SMOX, AMD1*
*Hypusine*	Hypusine biogenesis.	*DHPS, DOHH*
*Combined*	Empirically defined by negative correlation with CTL^a^ ssGSEA score across cancers.	*ODC1, SMS, SMOX, AMD1, ALDH18A1, PRODH, OAT, OAZ2, GPC1, SLC3A2, SLC7A1, SLC7A2*

^a^Cytotoxic lymphocyte gene set as defined in Bindea and colleagues.

**FIGURE 3:**
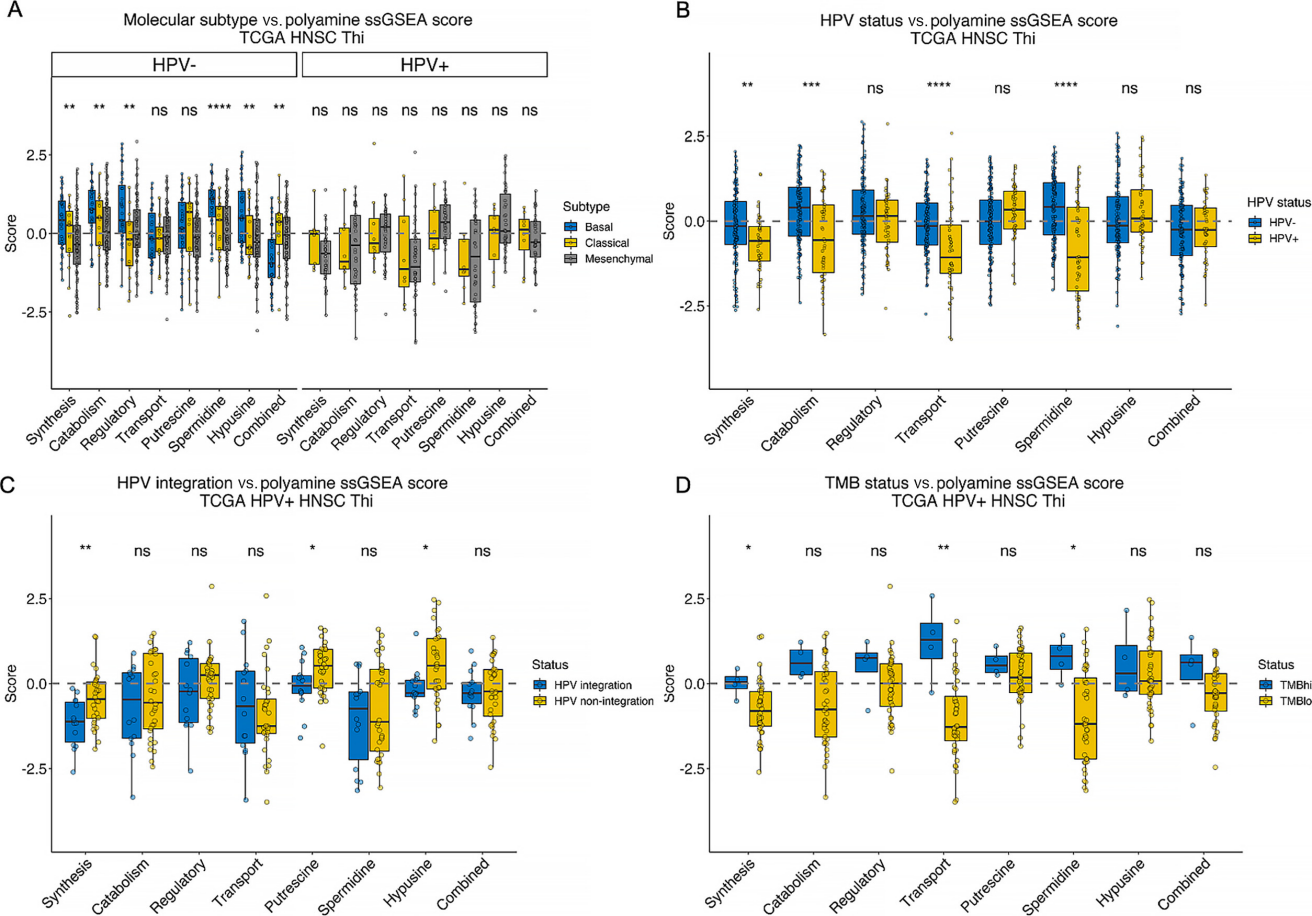
PA metabolism gene set expression and tumor-intrinsic features. PA gene set expression was quantitated using ssGSEA. PA metabolism genes were grouped into synthesis, catabolism, regulatory, transport, putrescine biosynthesis, spermidine biosynthesis, hypusine biosynthesis, and combined gene sets. PA ssGSEA scores across TCGA Thi HNSCs were stratified by molecular subtype (**A**), HPV status (**B**), HPV integration status (**C**), and TMB (*TMBhi*, ≥10 mutations/Mbp; *TMBlo*, <10 mutations/Mbp; **D**). *, *P* ≤ 0.05; **, *P* ≤ 0.01; ***, *P* ≤ 0.001; ****, *P* ≤ 0.0001; ns, *P* > 0.05.

Hypothesizing that PA metabolism gene expression depends on molecular subtype, we did not identify differences in enrichment of the PA gene sets among the HPV^+^ HNSCs (Fig. 3A). Among the HPV^−^ Thi HNSCs, ssGSEA scores from several PA gene sets were significantly higher among the basal compared with the classical or mesenchymal subtypes including the synthesis, catabolism, regulatory, spermidine, and hypusine gene sets. In contrast, PA-combined gene set ssGSEA scores were lower among the basal tumors compared with the classical or mesenchymal HPV^−^ HNSCs. HPV^+^ Tlo HNSCs also lacked differences in PA ssGSEA scores except for transport which was significantly higher among basal tumors whereas putrescine ssGSEA scores were higher among the classical and mesenchymal tumors than the basal tumors (Supplementary Fig. S5).

PA pathway expression was characterized on the basis of HPV status. Prior studies identified that viral replication depends on PAs (45) and that some viruses may stimulate PA synthesis (46, 47). Moreover, nicotine drives *ODC1* expression (48). PA synthesis, catabolism, transport, and spermidine ssGSEA scores were higher among HPV^−^ Thi versus HPV^+^ Thi HNSCs (Fig. 3B). PA synthesis and combined gene set ssGSEA scores were higher among HPV^−^ smokers compared with nonsmokers whereas regulatory ssGSEA scores were higher among the nonsmokers (Supplementary Fig. S6). HPV^+^ smokers demonstrated higher PA synthesis, transport, and combined ssGSEA scores than nonsmokers (Supplementary Fig. S6).

We examined whether HPV integration impacts PA pathway expression evaluating the association between PA pathway ssGSEA scores and HPV integration (18). Defining tumors as HPV integrated or HPV nonintegrated, we observed greater synthesis, putrescine, and hypusine ssGSEA scores among the HPV^+^ Thi HNSCs with nonintegrated HPV whereas there were no differences in the integration status among the HPV^+^ Tlo HNSCs (Fig. 3C; Supplementary Fig. S5C).

Finally, we tested the relationship between TMB and PA gene set expression given that PAs are involved in epigenetic regulation and DNA stabilization. Among HPV^+^ Thi HNSCs, PA synthesis, transport, and combined ssGSEA scores were higher among the TMBhi tumors (≥10 mutations/Mbp; Fig. 3D). TMBlo HPV^+^ Tlo HNSCs had higher regulatory ssGSEA scores than TMBhi HPV^+^ Tlo HNSCs (Supplementary Fig. S5D). Taken together, these data reveal evidence that PA gene set expression varies with molecular subtype, HPV status, integration status, and TMB among HPV^+^ Thi HNSCs. While these data reveal associations between PA gene set expression and tumor features, we were also interested in the extent to which tumor-infiltrating immune cells express PA genes. This could reveal insight into strategic targeting of PA metabolism to optimize antitumor immunity while diminishing tumor cell proliferation and viability.

### PA Gene Set Expression Among HNSC Intratumoral Immune Populations

**FIGURE 4:**
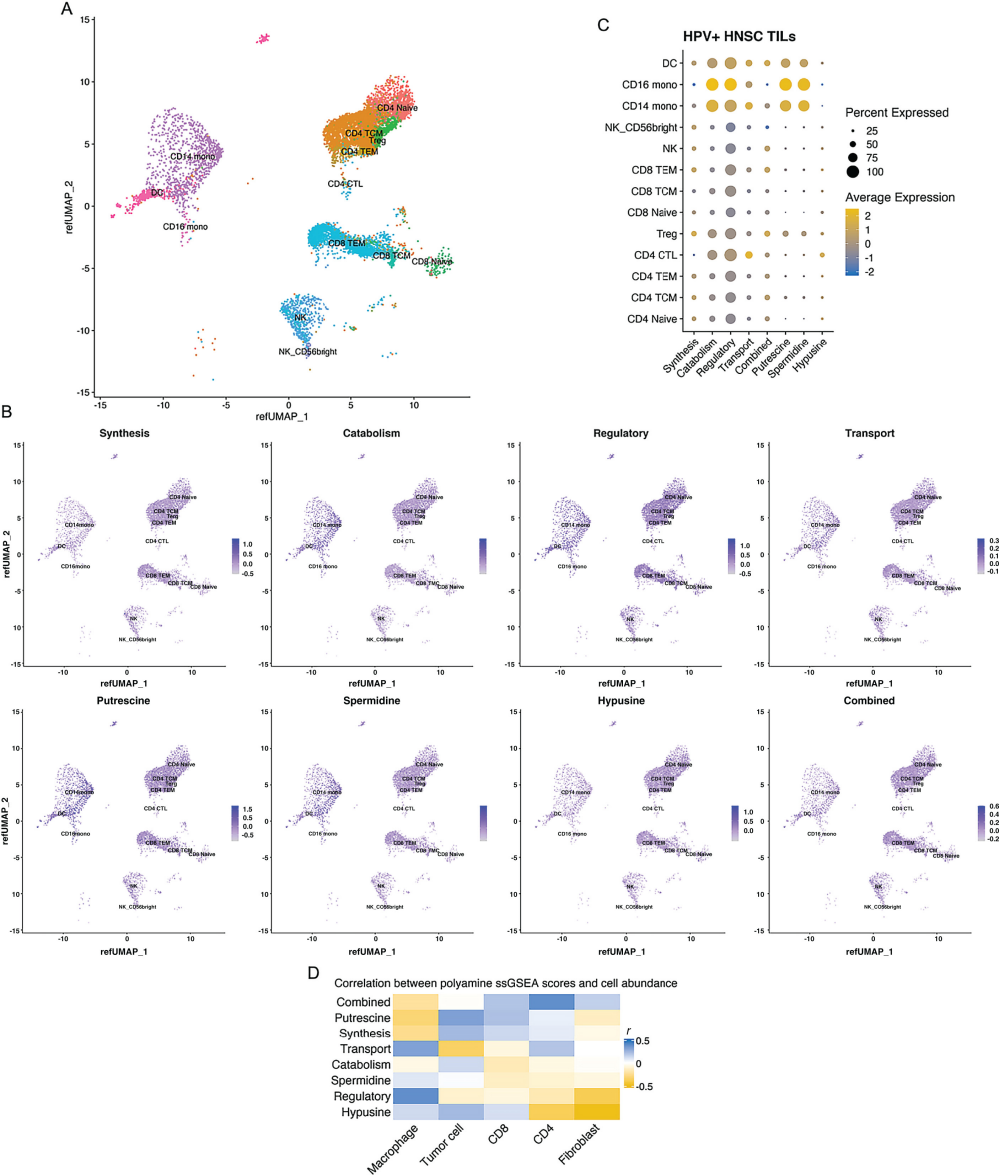
PA pathway expression and TILs in HPV^+^ HNSC. Publicly available TIL scRNA-seq data from eight HPV^+^ HNSCs (14,859 cells) were mapped onto a reference single-cell dataset to infer cell lineage. **A,** Uniform Manifold Approximation and Projection (UMAP) demonstrating lineage assignments across HPV^+^ HNSC TILs. **B,** PA metabolism pathway expression overlaid onto UMAP demonstrating pathway expression among clusters and quantified in **C**. **D,** CIBERSORT was used to infer cellular abundance for stromal cells in the TME using TCGA HPV^+^ Thi HNSCs. Cellular abundances were correlated with PA pathway ssGSEA scores and Pearson correlation coefficients (*r*) represented in the heatmap.

Given the importance of PA expression to T-cell and macrophage function (49, 50), we utilized scRNA-seq data to examine PA expression across HPV^+^ HNSC intratumoral immune populations (Fig. 4; ref. 21). We generated single-cell PA pathway enrichment scores using the gene sets defined above which we projected onto single cells (Fig. 4A–C). PA catabolism, regulatory, putrescine, and spermidine gene sets were significantly enriched in CD16^+^ and CD14^+^ monocytes relative to the lymphoid lineages (Fig. 4C; Supplementary Tables S12 and S13). PA transport gene set expression was enriched among the CD14^+^ monocytes relative to other lineages except CD4^+^ CTLs. PA synthesis gene set expression was significantly greater among CD4^+^ TCM, CD4^+^ naïve, CD8^+^ TEM, and regulatory T cells (Treg) relative to CD14^+^ monocytes. Expression of CD16^+^ and *SAT1*, *OAZ1/2*, *SMS*, and *SLC7A7* was differentially higher among CD14^+^ and CD16^+^ (except *SMS*) monocytes relative to other lineages (Supplementary Fig. S7; Supplementary Table S14). Moreover, analysis of scRNA-seq data from 21 HPV^−^ HNSCs (12) demonstrated greater PA regulatory, catabolism, and spermidine module expression in macrophages compared with T cells or tumors cells (Supplementary Fig. S8; Supplementary Tables S15 and S16). In contrast, HPV^−^ HNSC tumor cells had greater transport and combined pathway gene set expression than T cells, macrophages, or fibroblasts and higher synthesis gene set expression than T cells or fibroblasts (Supplementary Fig. S8; Supplementary Tables S15 and S16).

CIBERSORT was used to infer the cellular composition of the TME bulk RNA-seq data from TCGA HPV^+^ Thi HNSCs. These data were used to test the correlation between cell-type abundance and ssGSEA scores for PA pathway expression (Fig. 4D). After adjusting for multiple hypothesis testing, we observed a nonstatistically significant positive correlation between macrophage abundance and regulatory gene set expression (*r* = 0.39, FDR *q*-value = 0.29). Fibroblasts exhibited a strong negative correlation with hypusine gene set expression (*r* = −0.55, *q*-value = 0.003). Collectively, these data demonstrate relatively greater enrichment of PA catabolism and regulatory gene set expression in the myeloid compartment relative to the lymphoid compartment.

### Pan-Cancer PA Gene Set Expression and Immune Response

We tested the relationship between PA pathway gene set expression and T-cell receptor (TCR) diversity (10). We compared richness and Shannon entropy scores between PA gene set strata among TCGA HPV^+^ HNSCs. TCR richness and Shannon entropy were negatively associated with PA synthesis (Kruskal–Wallis test, *P* = 0.004 and *P* = 0.006, respectively; Supplementary Fig. S9). Other PA pathway gene set scores were not associated with TCR clonality among these tumors except putrescine and hypusine scores which were associated with higher richness and Shannon scores among the intermediate strata than the low or high putrescine and hypusine strata (Supplementary Fig. S9).

**FIGURE 5:**
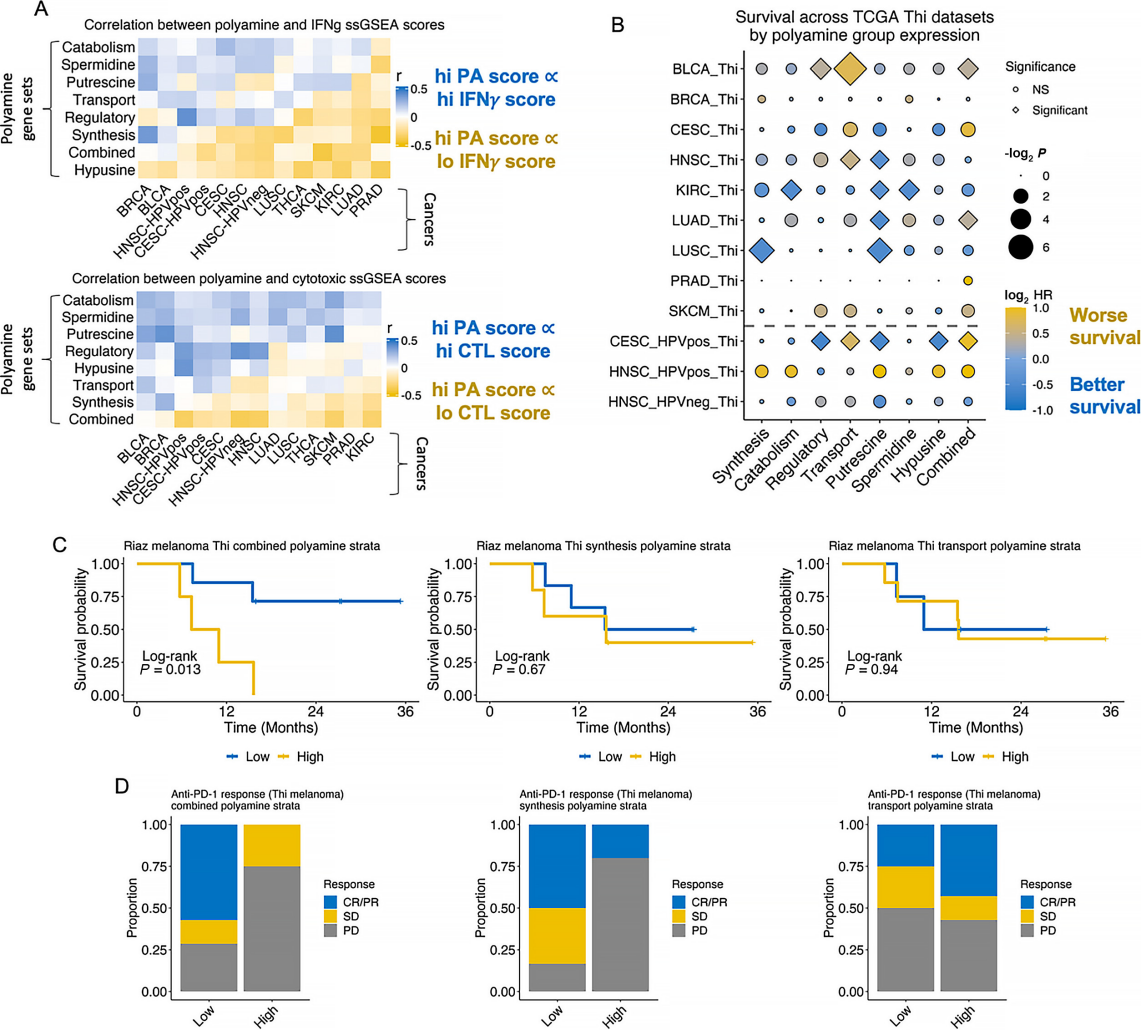
Immune functional markers and survival vary with PA pathway ssGSEA scores across T-infiltrated tumors. Ten of the mostly highly inflamed cancer types were stratified by T-cell infiltration status (Thi vs. Tlo) inferred using TCGA bulk RNA-seq data as described in the article. **A,** Correlation between PA ssGSEA scores and REACTOME IFNγ pathway (top) and cytotoxic lymphocyte pathway (bottom) ssGSEA scores are shown in the heatmaps. Correlation coefficients (*r*) for the correlation between PA ssGSEA score and immune pathway ssGSEA score for each cancer type are presented in the heatmap. **B,** Pan-cancer Cox regression analysis among the Thi tumors for each cancer type was performed using continuous PA ssGSEA scores as a covariate. log_2_ HRs are plotted. Diamonds represent statistically significant associations (*log test* − *log_2_ P,* FDR *q* < 0.25) and circles represent nonsignificant associations; size of shape represents magnitude of the *q*-value. **C,** Survival among nivolumab-treated Thi melanomas (*n* = 11) by combined (left; high: *n* = 4, low: *n* = 7), synthesis (middle; high = 5, low = 6), or transport (right; high: *n* = 7, low: *n* = 4) PA ssGSEA score strata. **D,** Barplots showing proportion of complete or partial responders (CR/PR), stable disease (SD), or progressive disease (PD) given combined (left), synthesis (middle), or transport (right) PA ssGSEA score strata.

We further hypothesized that tumor cell PA synthesis and/or transport gene set expression is associated with worse antitumor T-cell function as inferred by IFNγ or cytotoxic lymphocyte ssGSEA scores. Correlation analyses were performed between the REACTOME IFNγ signaling gene set or the Bindea CTL ssGSEA scores and PA pathway ssGSEA scores (Fig. 5A). The IFNγ ssGSEA scores were significantly negatively correlated with PA combined, synthesis, and hypusine ssGSEA scores in seven (*r* range: −0.42 to −0.17), six (*r* range: −0.45 to −0.25), and 12 (*r* range: −0.38 to −0.15) cancer types, respectively (FDR *q* < 0.25). IFNγ scores were positively correlated with catabolism or putrescine scores in four cancer types, respectively. Similarly, CTL gene set scores were significantly negatively correlated with PA combined pathway scores in nine cancer types (*R* range: −0.39 to −0.16) and positively correlated with PA catabolism and regulatory scores in 10 (*R* range: 0.12–0.29) and six (*R* range: 0.18–0.37) cancer types (Fig. 5A). Hypothesizing that PAs contribute to immunosuppression, we sought to characterize the association between PA pathway ssGSEA scores and survival across cancers.

### Pan-Cancer PA Gene Set Expression and Outcomes in T Cell–enriched Tumors

We performed Cox regression testing the association between PA pathway ssGSEA scores and risk of mortality in T cell–enriched tumors. High transport scores were associated with a greater risk of mortality among Bladder urothelial carcinoma (BLCA), HNSC, and HPV^+^ Cervical and endocervical cancers (CESC) TCGA cohorts. Combined PA pathway gene set scores were associated with higher mortality among the BLCA, Lung adenocarcinoma (LUAD), and HPV^+^ CESC cohorts, with a trend toward worse survival among the Prostate adenocarcinoma (PRAD), Skin Cutaneous Melanoma (SKCM), HPV^+^ HNSC and CESC tumors (Fig. 5B). Interestingly, high putrescine ssGSEA scores were associated with better survival among HNSC, KIRC, LUAD, LUSC, and HPV^+^ CESC Thi tumors. In contrast, PA combined pathway gene set scores were not associated with increased risk of mortality across Tlo tumors (Supplementary Fig. S10). However, higher PA transport scores were associated with worse survival among Tlo HNSC, SKCM, and BLCAs.

Given the negative association between the PA combined pathway ssGSEA scores with effector lymphocyte gene set scores and prognosis, we hypothesized that PA expression affects response to ICI. Specifically, we were interested in the association with ICI response based on PA biosynthetic and transport pathway expression (given the availability of commercially available inhibitors of these functions) among a subset of T cell–enriched, nivolumab-treated melanomas (26). High combined PA pathway ssGSEA scores were associated with worse survival (Fig. 5C). There was no difference in survival based on the synthesis or transport ssGSEA scores. Response to ICI was greatest among patients with low combined or synthetic gene set ssGSEA scores (Fig. 5D).

## Discussion

To identify metabolic features impairing the antitumor immune response in an otherwise favorable TME, we leveraged T cell–infiltrated, immunogenic, HPV^+^ HNSC. Using this strategy, PA gene set expression was associated with aggressive molecular phenotypes, diminished antitumor immunity, poor prognosis across cancer types, and a poor response to immunotherapy among melanomas. These data demonstrated both tumor-dependent and stromal differences in PA metabolism gene expression. As PAs are necessary for tumor proliferation and function, T-cell activation and differentiation, and macrophage differentiation, the PA metabolism axis represents a therapeutic target for leveraging divergent PA metabolic needs between tumor and immune cells.

PAs, a family of low molecular weight polycations, regulate cell processes from proliferation and adaptive immunity (51, 52) to epigenetic modifications (53, 54), metabolite availability (55, 56), transcriptional regulation (57), and chromatin stabilization (58). Cells primarily synthesize PAs and acquire them from the TME. Given the essential role of PAs in tumor and T-cell function (49), this pathway may be rationally targeted to diminish tumor growth and/or enhance antitumor immunity. In recent work, investigators identified the dependence of CD4^+^ T-cell differentiation on PA metabolism, for example demonstrating that T_H_17 cells and T_regs_ may rely more on transport than PA synthesis (59, 60). In our scRNA-seq analyses among tumor TILs from HPV^+^ HNSCs, we did not observe a difference in PA transport gene set expression between T_regs_ and CD4^+^ CTL, CD4^+^ T_CM_, or CD4^+^ T_EM_ cells (Supplementary Table S13). In contrast, we identified relatively greater PA spermidine and putrescine gene set expression among the T_regs_ compared with CD4^+^ T_CM_ and CD4^+^ T_EM_ cells. We did not test PA gene set expression in other CD4^+^ T-cell lineages as this was beyond the scope of this study, but it would be intriguing to compare the effect of PA metabolism on CD4^+^ T-cell differentiation in the context of the TME.

The metabolic milieu of the TME diminishes antitumor immune control by producing inhibitory metabolites, depleting essential nutrients, and creating a hypoxic and acidic ecosystem. Here, PA gene set expression was upregulated in high-risk, T cell–infiltrated HPV^+^ HNSCs. Gene expression clusters with T cell–infiltrated tumors were enriched in PA catabolism and regulation genes (i.e., *SAT1*, *OAZ1*–3). PA gene expression varied by molecular subtype, HPV status, HPV integration status, and TMB status. High TMB HPV^+^ Thi HNSCs were associated with higher PA synthesis, transport, and spermidine gene set scores. At the single-cell level in HPV^+^ HNSCs, PA catabolism, regulatory, transport, putrescine, and spermidine gene set ssGSEA scores were enriched in the myeloid compartment relative to lymphoid lineages. In the pan-cancer analysis, we observed negative correlations between PA synthesis, transport, combined, or hypusine gene set ssGSEA scores with effector lymphocyte function ssGSEA scores. The question persists of whether immunosuppressive immune populations leverage PA metabolism to permit tumor growth and whether there is a feedback loop of PA-dependent tumor-intrinsic paracrine function promoting immunosuppressive immune function.

PA blockade therapy (AMXT1501/DFMO) using difluoromethylornithine (DFMO), an ornithine decarboxylase inhibitor, and AMXT1501 (31, 61), a PA transport inhibitor, demonstrates excellent responses in diffuse intrinsic pontine glioma (62) or *MYCN* transgenic mice models (31). AMXT1501/DFMO shows activity in immunocompetent, but not immunodeficient mouse tumor models (63). In contrast, monotherapy is less effective than combined therapy, likely secondary to compensatory mechanisms. This may account for the limited responses noted to date with PA monotherapy (64, 65). In is intriguing to speculate that PA expression in the TME may serve as a biomarker for patient selection in employing agents of the PA pathway such as in the case of patients with HPV^+^ HNSC with increased T-cell infiltrates and high PA levels.

Disruption of PA homeostasis in the TME may affect the function of infiltrating immune cells. Several metabolites contribute to PA synthesis including methionine, glutamine, arginine, and proline. About 30% of PAs are derived from glutamine in activated T cells, though PA synthesis blockade prevents T-cell expansion (49). In macrophages, inhibition of either PA synthesis or hypusination diminishes oxidative phosphorylation and prevents alternative macrophage differentiation (50). PAs are immunosuppressive, and tumors may leverage PAs for intrinsically mediated immune evasion by inducing autophagy (66), favorable epigenetic alterations (53), or stabilizing their DNA from the effects of cytotoxic lymphocytes (58).

Future studies will need to address some of the limitations of this study including the small sample size of HPV^+^ HNSCs, limiting our power for survival analyses. Cohorts powered to detect differences in survival as a function of metabolite enrichment may further elucidate mechanisms diminishing antitumor immunity. Defining the extent to which PA metabolism diminishes antitumor immunity either because of exposure to supraphysiologic intracellular PA levels and/or through cell-intrinsic mechanisms that promote tumor cell survival will help overcome hurdles to immunotherapy. Finally, the role of PA transport in T-cell function is unknown. The degree to which putative PA transporters regulate nutrient transport and antitumor immunity requires further investigation. These data aim to uncover fundamental insights into the effect of metabolites in the TME and antitumor immune responses and spur a line of inquiry investigating the extent to which PA metabolism impairs antitumor immunity.

## Supplementary Material

Supplementary Tables S1-S17 Supplementary Tables

Supplemental Figures S1-S10 Supplemental Figures S1-10
