# Supplementary material for: Interrogation of T Cell–enriched Tumors Reveals Prognostic and Immunotherapeutic Implications of Polyamine Metabolism
Source: Cancer Res Commun. 2022 Jul 13;2(7):639–52. doi: 10.1158/2767-9764.CRC-22-0061 (PMC9432485; doi:10.1158/2767-9764.CRC-22-0061)
Supplement: Supplemental Figures S1-S10 — Supplemental Figures S1-10 [file crc-22-0061-s02.pdf]

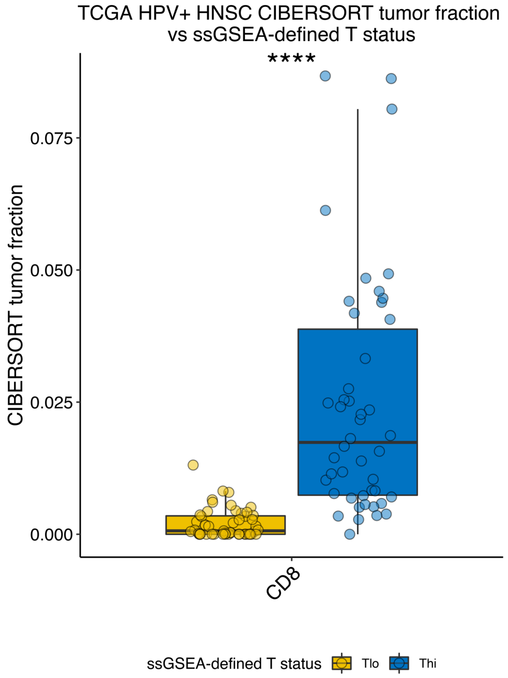

Figure S1. CIBERSORT relative CD8⁺ T cell tumor fraction versus ssGSEA-defined T cell infiltration status. CD8⁺ T cell infiltration as a proportion of cells in the tumor microenvironment inferred from bulk RNAseq data using CIBERSORT is stratified by ssGSEA-defined T cell infiltration status among HPV+ HNSCs. ****, $p \leq 0.0001$.

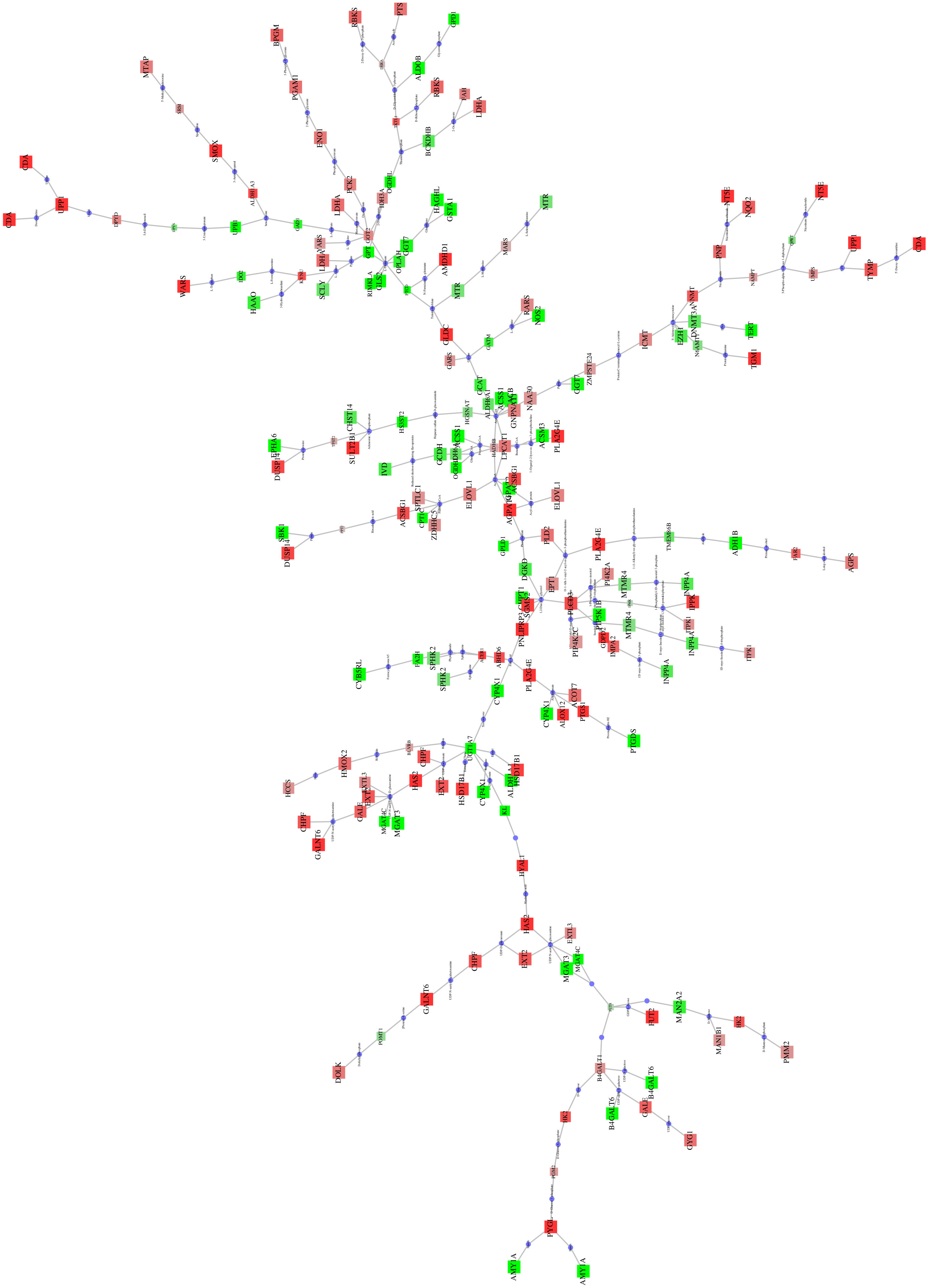

Figure S2. Metabolic gene transcriptional network analysis comparing TCGA HPV+Thi HNSCs based on the high-risk HPV+ HNSC C1 gene set differential expression results (C1 high vs C1 low). *Red nodes*, gene expression enriched in C1 high tumors. *Green nodes*, gene expression enriched in C1 low HPV+ HNSCs.

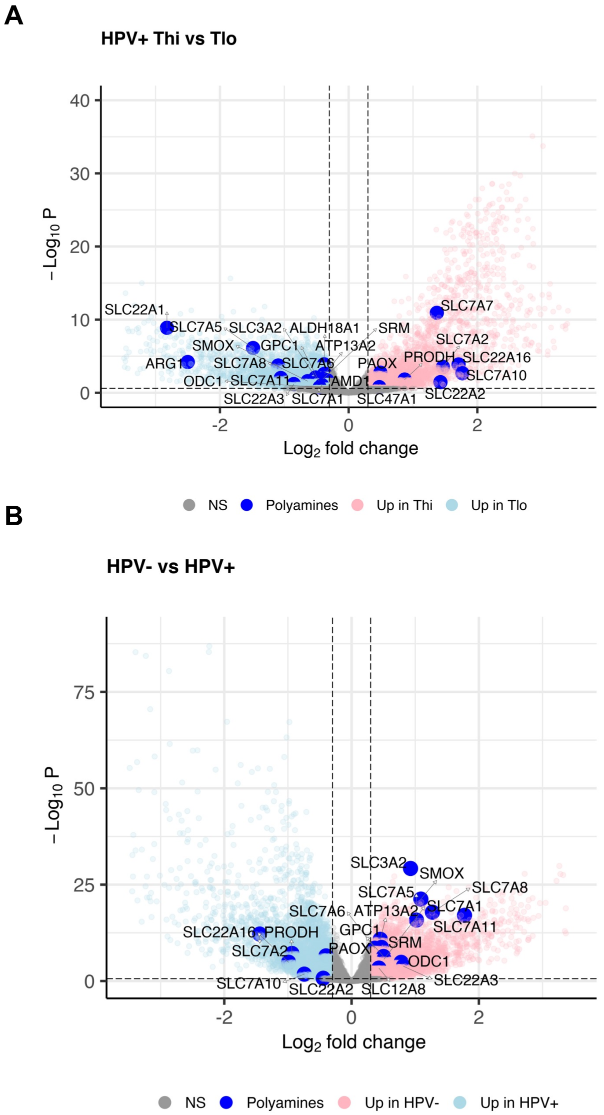

Figure S3. Differential expression analyses of TCGA HNSC RNAseq data. (**A**) HPV+ Thi vs Tlo HNSC. *Pink circles*, gene upregulated in Thi tumors. *Light blue circles*, genes upregulated in Tlo tumors. (**B**) HPV+ vs HPV- HNSC. *Pink circles*, gene upregulated in HPV- tumors. *Light blue circles*, genes upregulated in HPV+ tumors. *Dark blue circles*, polyamine pathway genes labeled.

A

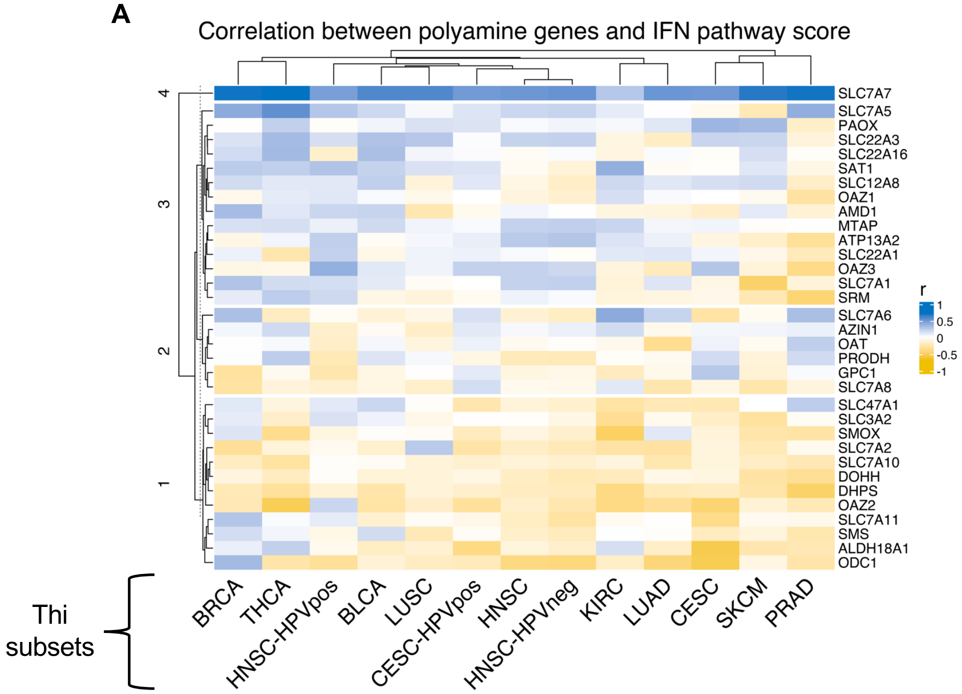

B

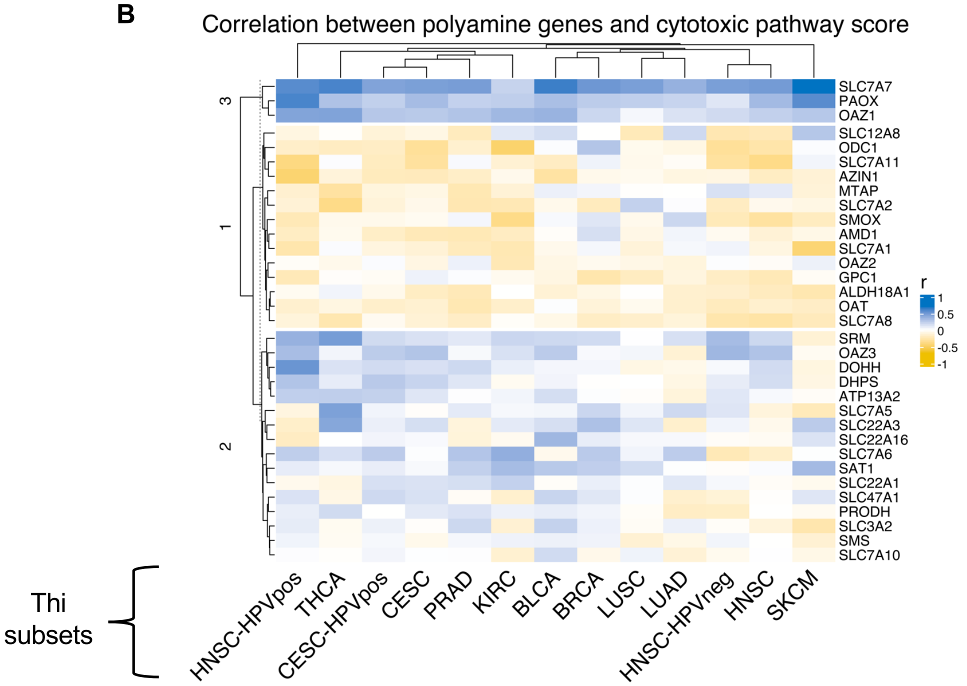

Figure S4. Correlation between polyamine metabolism gene expression and ssGSEA scores for IFNγ and cytotoxic lymphocyte gene sets across TCGA Thi tumors. (**A**) Results from pancancer analysis of correlation between polyamine gene expression and IFNγ gene set ssGSEA score. (**B**) Results from pancancer analysis of correlation between polyamine gene expression and cytotoxic lymphocyte ssGSEA score. *Blue,* positive correlation. *Gold*, negative correlation. Rows represent genes. Columns represent cancer type. Individual rectangles represent correlation coefficients for the correlation between gene expression and ssGSEA score across cancers.

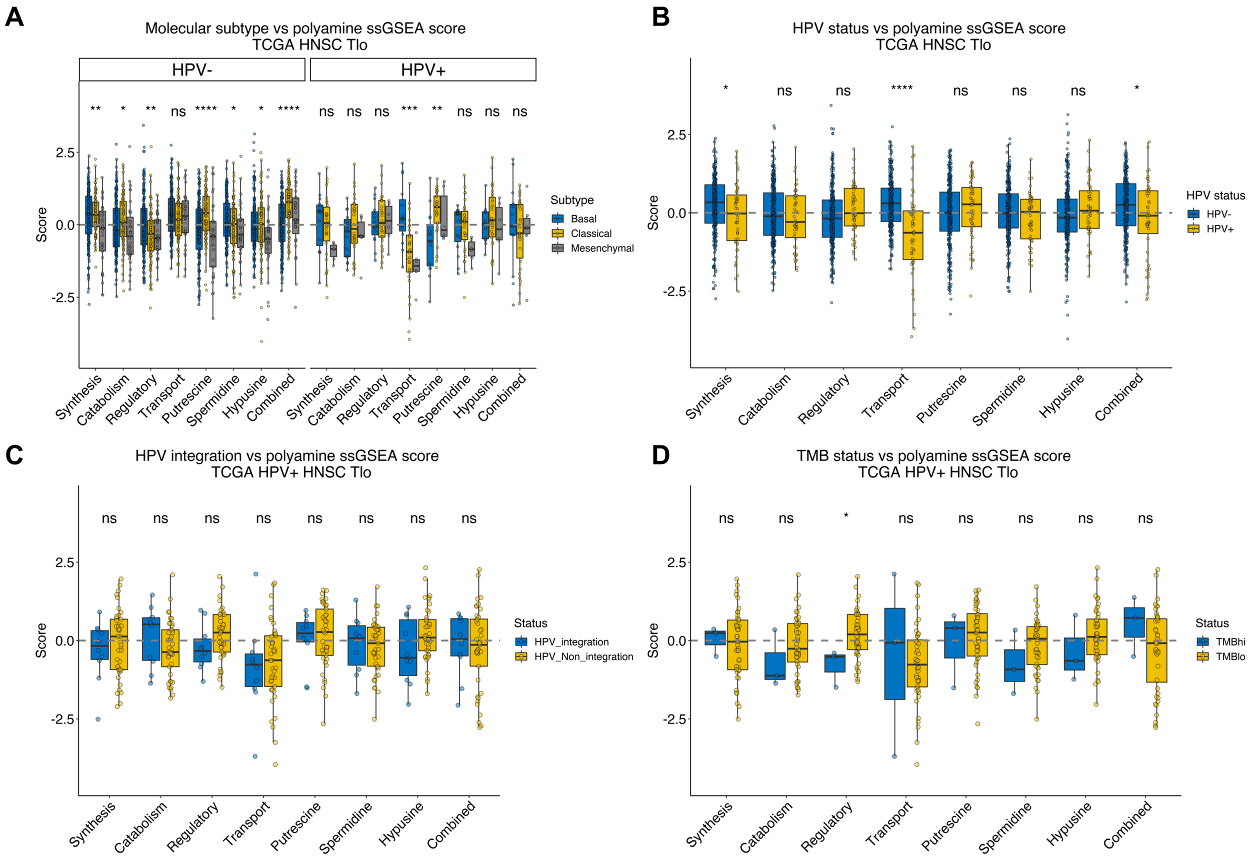

Figure S5. Polyamine metabolism gene expression and tumor-intrinsic features among TCGA Tlo HNSCs. Polyamine gene expression grouped by functional category and ssGSEA performed to generate scores. Polyamine gene set scores across TCGA Tlo HNSCs stratified by **(A)** molecular subtype, **(B)** HPV status, **(C)** HPV integration status as defined in Parfenov et al., and **(D)** tumor mutation burden (*TMBhi*, ≥ 10 mutations/Mbp; *TMBlo*, < 10 mutations/Mbp). *, p ≤ 0.05; **, p ≤ 0.01; ***, p ≤ 0.001; ****, p ≤ 0.0001; ns, p > 0.05.

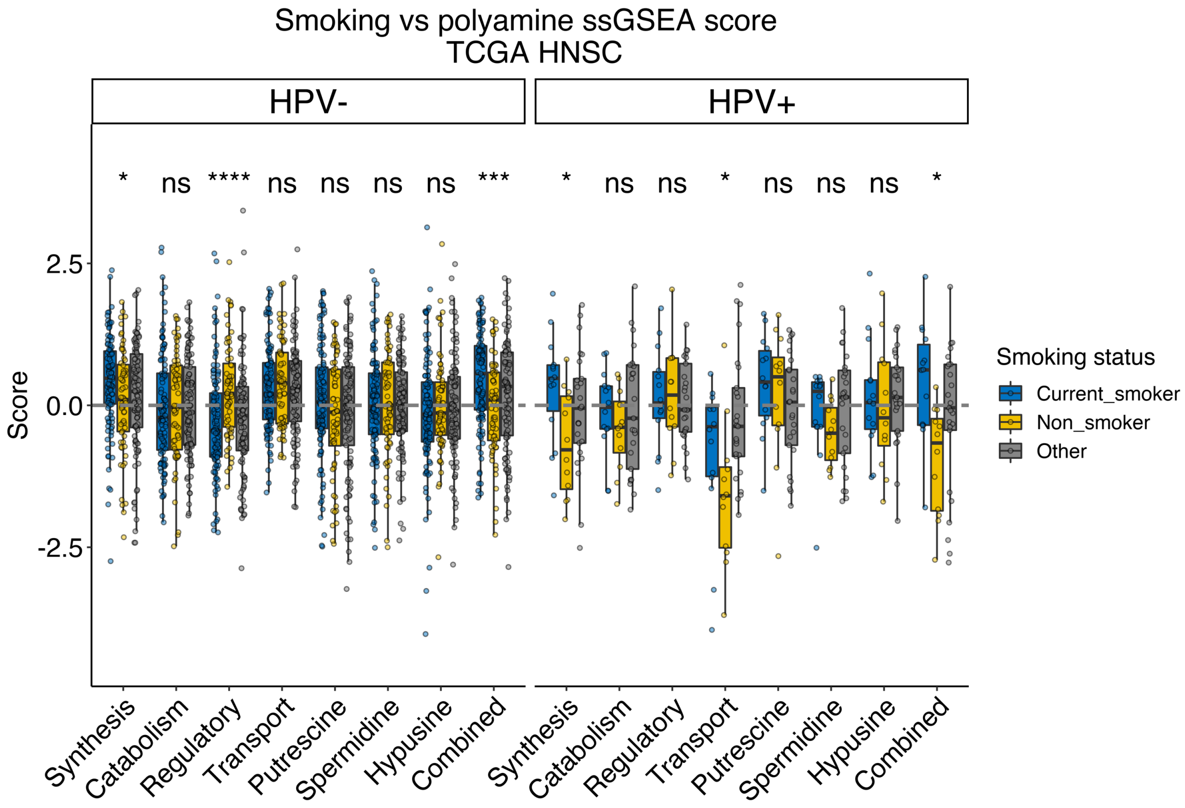

Figure S6. Polyamine metabolism gene expression and smoking among TCGA HPV+ vs HPV- HNSCs. Polyamine gene expression grouped by functional category. Polyamine ssGSEA scores are stratified by smoking status and plotted for HPV- and HPV+ HNSCs. *, $p \leq 0.05$; ***, $p \leq 0.001$; ****, $p \leq 0.0001$; ns, $p > 0.05$.

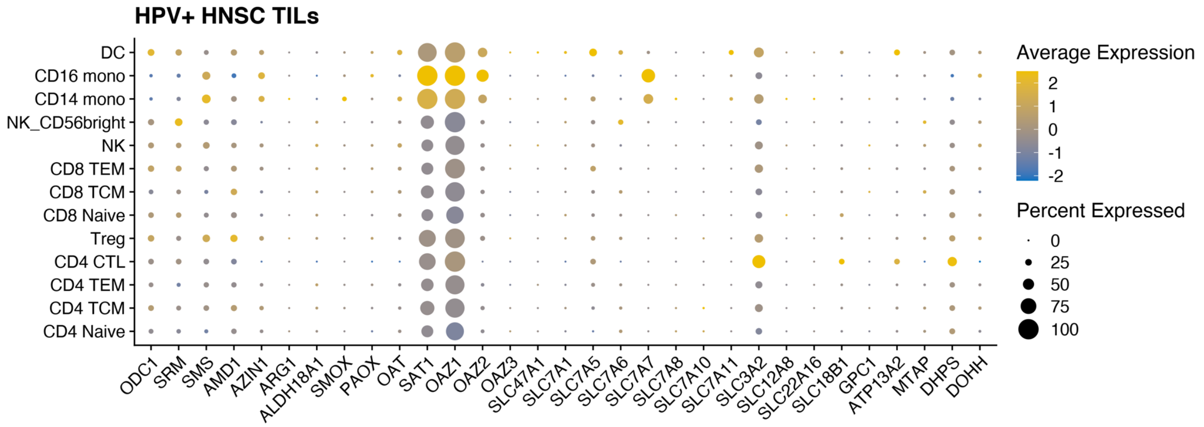

Figure S7. Polyamine metabolism gene expression among tumor infiltrating lymphocytes in the HPV+ HNSC tumor microenvironment. TIL single cell RNA sequencing data (Cillo et al.) from eight HPV+ HNSCs (14,859 cells) were mapped onto a reference single cell data set to infer cell lineage. Gene expression is plotted among inferred cell lineages. Scaled expression and percent of cells expressing each gene are represented on plot.

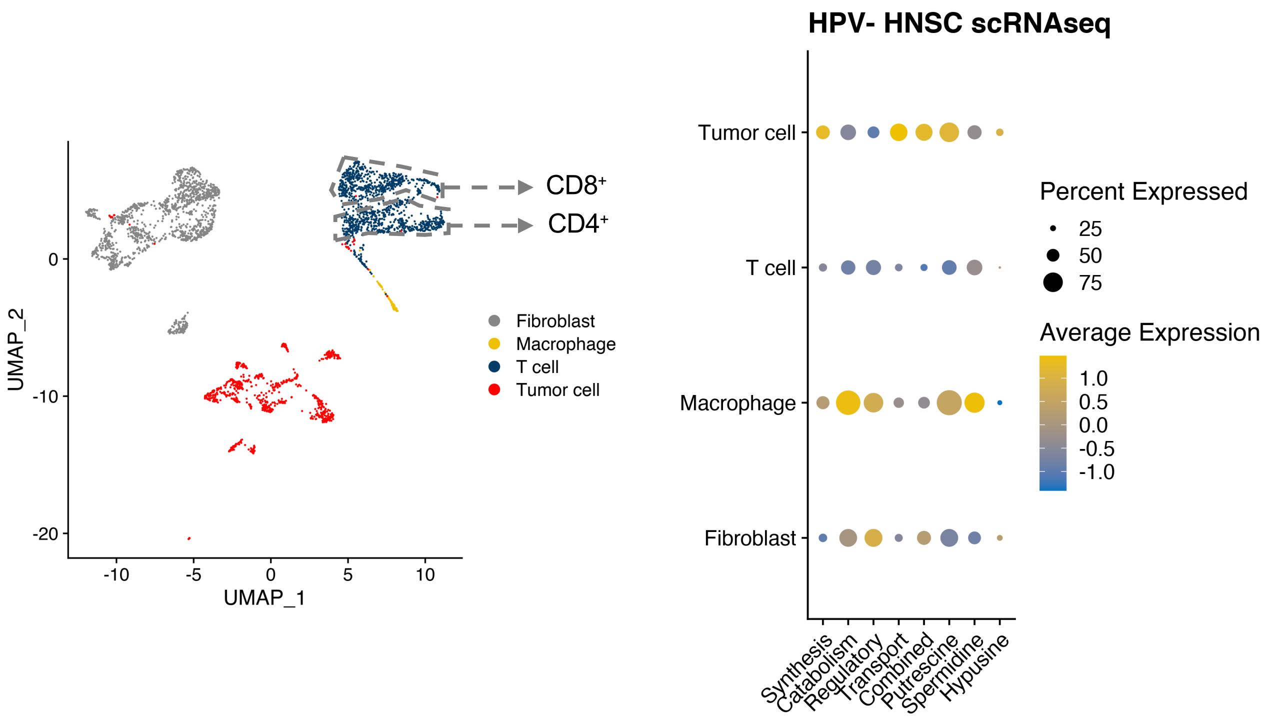

Figure S8. Polyamine module expression in the HPV- HNSC tumor microenvironment. (**A**) UMAP of single cell RNA sequencing data (Puram *et al*) from 21 HPV- HNSC tumor samples (3,478 cells) with cell lineage labeled according to the original classification. (**B**) Polyamine metabolism gene module expression was calculated and quantified by cell lineage.

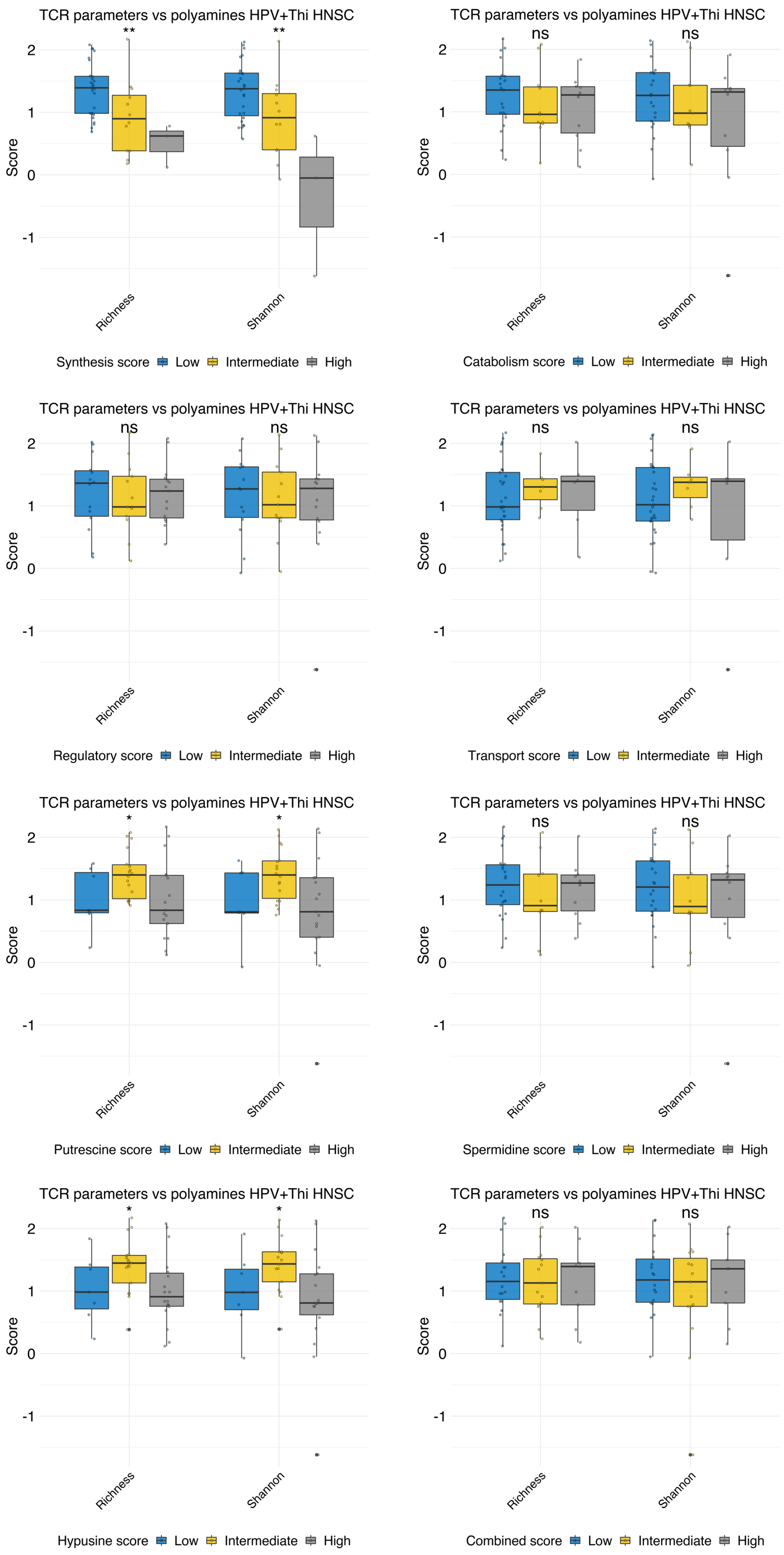

Figure S9. Polyamine metabolism gene expression vs T cell receptor clonality richness and Shannon entropy among TCGA HPV+Thi HNSC. Richness and Shannon scores from Thorsson et al. among TCGA HPV+Thi HNSCs (n = 97) were used. Stratified polyamine metabolism ssGSEA scores were used to assess for a relationship with TCR clonality. **, p ≤ 0.01.

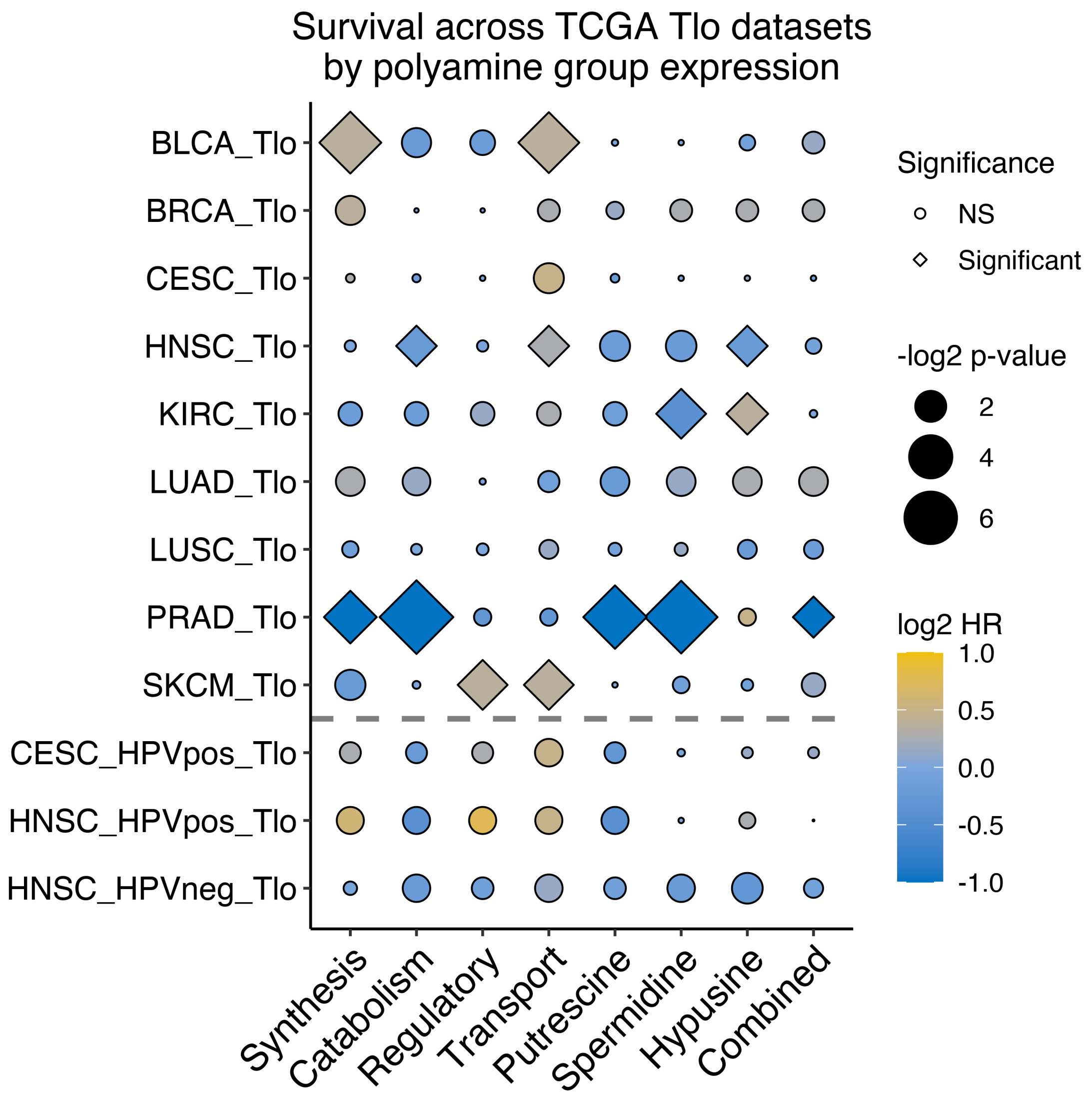

Figure S10. Survival versus polyamine pathway gene set scores across T cell-depleted cancers. Pan-cancer Cox proportional hazards analysis among the Tlo tumors for each cancer type was performed using continuous polyamine ssGSEA scores as a covariate. Log2 hazard ratios (HR) are plotted. Diamonds represent statistically significant associations (*Log test -log2 p-value,* FDR q < 0.25) and circles represent non-significant associations; size of shape represents magnitude of the q-value.
